# Improving the Fracture Toughness and Ductility of Liquid-Phase Sintered WNiFe Tungsten Heavy Alloys by High-Temperature Annealing

**DOI:** 10.3390/ma16030916

**Published:** 2023-01-18

**Authors:** Md Ershadul Alam, G. Robert Odette

**Affiliations:** Materials Department, University of California, Santa Barbara, CA 93106, USA

**Keywords:** tungsten heavy alloy, high-temperature heat treatment, annealing, microstructure, fracture toughness, ductile phase toughening

## Abstract

Tungsten heavy alloys (WHAs) are candidates for use in fusion reactor divertors. Here, we characterize liquid-phase sintered WHAs with 90, 92.5, 95, and 97 (wt.%) tungsten (W), with a balance of a 0.7Ni–0.3Fe ductile phase. These WHAs show remarkable room temperature (RT) fracture toughness at the maximum load, K_Jm_, ranging from ≈ 38 to 107 MPa√m, compared to a monolithic W toughness of ≈ 8 MPa√m. In most cases, the fracture of WHAs occurs through stable crack tearing. However, the 97W WHA has the lowest toughness and fracture elastically in all but the smallest specimens. As lower Ni contents are desirable for fusion application, we explore the potential for improving the ductility and K_Jm_ of WHAs using vacuum annealing at 1300 °C for 24 h. The microstructural observations reveal negligible changes in the WHA microstructure and constituent compositions. While annealing reduces the Vickers microhardness (HV), it does not significantly change the RT yield (σ_y_) and ultimate (σ_u_) strengths but results in beneficial increases in total elongation in the 95 and 97W WHAs by a factor of 2. RT tests on the precracked three-point-bend (3PB) bars show that annealing increases the K_Jm_ of these WHAs, and in the case of the 97W WHA, the increase is from 42 to 92%, depending on the size of the specimen. Toughening is due to enhanced crack tip process zone microcracking and dilatation.

## 1. Introduction

Tungsten heavy alloys (WHAs) are a class of bi-phasic metallic composites composed of tungsten (W) powders consolidated by liquid-phase sintering (LPS) with lower melting point ductile phase (DP) metals or alloys consisting of Ni, Fe, Cu, and Co constituents. WHAs are known for their good room- to high-temperature tensile strength and ductility [1,2,3,4]. In addition, for use in very high temperatures environments, such as rocket nozzles, the applications of WHAs include ordnance, such as kinetic energy penetrators, counterbalances, and flywheels, where high mass densities are needed [1,3,5]. WHAs are now considered one of the most promising plasma-facing structural materials for fusion reactor divertor and armor applications [6,7,8,9,10,11,12,13]. For example, Neu et al. [8,9] found that 97W-2Ni-1Fe WHAs divertor tiles in the mid-size tokamak ASDEX upgrade facility that experienced up to a 20 MW/m^2^ cyclic plasma heat flux and up to 2200 °C surface temperatures, showed a lower cracking tendency compared to monolithic W. The advantages and open questions about WHAs related to fusion service are described elsewhere [7,9,11,12,13,14,15], and the WHA fabrication routes and basic properties are the subjects of extensive literature [1,2,3,4,5,15,16]. For various reasons, such as W recrystallization and fuzz formation, the maximum service temperatures for W divertors are likely to be ≈1300 °C [8,9,17,18,19,20,21,22].

Sufficient fracture toughness is critical to qualify a structural material for fusion divertor applications. Our literature search shows no studies on the use of high-temperature annealing heat treatments to improve the fracture toughness of as-sintered WHAs. Indeed, fracture toughness evaluations on the precracked WHA specimens for any compositions or conditions following ASTM standards is very rare. Note, others work on the heat treatment on WHAs mostly involves the impurity phase or tensile property characterization and can be found in [23,24,25,26]. Here, we characterize the microstructure, tensile properties, and most importantly, the elastic plastic fracture toughness of the WHAs. Our previous studies characterizing 90–97W WHAs showed that the maximum load fracture toughness (K_Jm_) was similar in alloys with 90 to 95W but was lower at 97W [14]. As lower Ni contents and higher K_Jm_ are desirable, we explore the potential for improving the K_Jm_ of a series of WHAs, including a lower Ni content 95 and 97W, using vacuum annealing at 1300 °C for 24 h.

The detailed microstructural observations show annealing has little effect on the WHA microstructure—W particle size, contiguity, DP phase compositions, area fraction, and thickness. Note, unless otherwise stated, here, annealing (AN) indicates a 1300 °C/24 h condition. Vickers microhardness (HV) was used to probe individual W, the ductile phases, and the WHA composite. Annealing decreases HV in all cases, especially at higher W contents. The RT tensile 0.2% yield strength (σ_y_) of all the WHAs and the total elongation (ε) of the 90 and 92.5W WHAs were only weakly dependent on annealing, with the most significant effect for the 97W. However, annealing the 95 and 97W alloys nearly doubles their respective tensile ductility.

The as-sintered (AS) RT fracture toughness of 3PB bars with nominal length/width/thickness dimensions of ≈ 16/3.3/1.65 mm (with a designated size of 1×) to 110/25.4/12.7 mm (8×) [27]. Note, there is a significant K_Jm_ decrease between the 1× and 3× specimens (up to 45% drop), with minimal K_Jm_ variations between the 3× and 8× specimens (less than 5%) [27]. Note, the ASTM E1921 standard was used to evaluate the maximum load elastic K_Im_ and elastic–plastic K_Jm_ toughness [28]. Annealing was carried out on the 1× and 4× (60/12.7/6.35 mm) 90–97W specimens. Annealing improved the RT K_Jm_ of all WHAs, especially the 97W alloy, in this case by ≈42%. The enhanced toughening is primarily due to enhanced process zone microcracking and dilatation. Annealing results in an increased amount of more widely distributed microcracking. Annealing also reduced the high fraction of cleavage fracture peculiar to the 97W AS condition. Finally, a lower temperature and shorter time anneal of 1100 °C/1 h (labeled 111) and 1200 °C/1 h (labeled 121) did not affect K_Jm_ but slightly improved K_Im_ compared to the AS 3× 97W condition.

## 2. Materials and Methods

Details of material acquisition, specimen fabrication, microstructural observation, precracking, and mechanical testing procedures can be found elsewhere [14,27]. Briefly, four commercially available 90, 92.5, 95, and 97 wt.% W, with a balance of DP (Ni/Fe = 7/3 ratio), are described as 90W, 92.5W, 95W, and 97W, respectively. Table 1 summarizes the compositions and densities of these WHAs. The WHAs were acquired from Mi-Tech Metals, Indianapolis, IN, USA, as liquid-phase sintered plates. The electrical discharge machining (EDM)-fabricated AS specimens were ground using 220 to 2000 grit silicon carbide sandpaper to remove any EDM damage, residual stress, and surface oxides, followed by 10 min ultrasonic vibration clean in acetone and testing for as-sintered (AS) condition. Acetone-cleaned molybdenum sheet (purity > 99.95%) on an alumina tray (purity > 99.8%) was used to house some of these cleaned AS specimens in a vacuum chamber during annealing in a vacuum resistance furnace. All the specimens were annealed (AN) at 1300 °C for 24 h, except two 4× 97W that were annealed at 1 h for 1100 and 1200 °C. A 5 °C/min heating and cooling rate was used.

Microstructural characterization, including optical, scanning electron microscopy (SEM), energy dispersive spectroscopy (EDS), and electron backscatter diffraction (EBSD) was carried out on the pre-and post-annealed conditions. The W-particle diameter (D_w_), W-W contiguity, DP composition, DP area fraction, ligament thickness (t), ligament thickness to W-particle diameter ratio (t/D_W_), and local fracture modes were observed and analyzed using imageJ64 software. Vickers microhardness (HV) was probed on both individual W and ductile phases at low load (10_gf_) and the composites at 500_gf_ load, both for the AS and AN conditions. Uniaxial tensile tests at RT were carried on EDM-fabricated flat dog-bone-shaped sub-sized SSJ2 specimens with a nominal gauge section length/width/thickness dimension of 5.0/1.2/0.5 mm [14,29]. A servo-hydraulic (model: MTS 810) universal testing machine was used to perform the tensile test at a strain rate of 10^−3^/s in accordance with the ASTM E8 standard [30].

The RT fracture toughness tests on the WHAs were conducted on fatigue precracked 3PB bars, with nominal dimensions (length/width/thickness) of 1× ≈ 16/3.3/1.65 mm, 3× ≈ 50/10/5 mm, and 4× ≈ 60/12.7/6.35 mm. All the specimens were fatigue-precracked to a/W ≈0.4 to 0.5 at 20 Hz at a maximum ∆K_I_ ≈ 15 MPa√m and a load ratio R ≈ 0.1. The bend test load (P) and load-point displacements (d) were measured, and K_Jm_ was defined as the maximum load (P_m_) based on the ASTM E1921 standard practice method of estimating the J-integral elastic–plastic fracture toughness: J_m_ = J_e_ + J_p_; K_Jm_ = √{EJ_m_/(1−ν^2^)}; J_e_ = K_Im_^2^(1−ν^2^)/E. Here, K_Im_ is the elastic stress intensity factor at maximum load, J_p_ = 2A_P_/Bb_o_ [28], B is specimen thickness, b_o_ is the initial unbroken ligament dimension, and A_p_ is the plastic area under the load-displacement curve [28,31,32]. It is also of interest to compare normalized P/P_o_ –d/S curves by dividing P by the plane strain limit load P_o_ [27,31] and d by the bend bar span, S. It is important to note that most tests that were not interrupted shortly beyond the maximum load involved at least some ductile tearing. Further details of toughness characterization procedures are reported in [27]. At least four specimens were characterized for 1× AS and AN and 3× AS conditions, and two specimens for 4× AN conditions. Additionally, two of the 4× 97W specimens were annealed at 1100 and 1200 °C/1 h and were tested at RT.

## 3. Results

### 3.1. Microstructural Characterization

**Figure 1:**
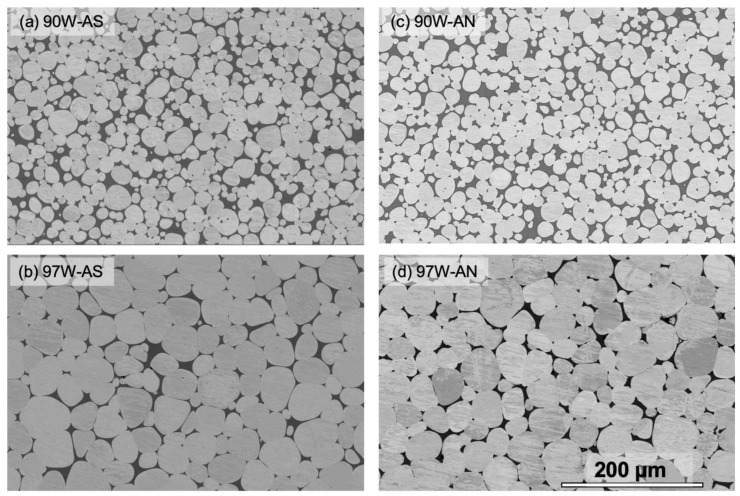
Low magnification SEM images of 90 and 97W WHAs: (**a**,**b**) before annealing; (**c**,**d**) after annealing. The scale bar is 200 µm for all cases.

Figure 1 shows the SEM micrographs of the polished and etched 90 and 97W WHAs before (Figure 1a,b) and after (Figure 1c,d) annealing. The SEM micrographs of the W particles and DP morphology for all four alloys are shown in Supplementary Materials, Figure S1. The microstructural observation results are summarized in Table 2. In all cases, the W particles are roughly spheroidal, surrounded by an interconnected honeycomb web of DP. The multiple-point EDS scans reveal that the particles are nearly 100% W, irrespective of the alloy compositions or annealing conditions. The size of the W particles does not change before and after annealing but increases with the W content: ≈ 17 ± 7 µm for 90W to ≈ 38 ± 15 µm for 97W, as shown in Table 2 and Figure 1. The DP fraction (%) is also unaffected by annealing. The multiple-point EDS scans on the ductile phase show that the DP composition is also unaffected by annealing, averaging ≈ 52% Ni, 31% W, and 17% Fe (AN) versus 50% Ni, 32% W, and 18% W (AS) by wt., as shown in Supplementary Materials, Table S1 and Figure S2. The W–W contiguity increases with W but remains unchanged after annealing for the 90 to 95W; it appears to decrease slightly for the 97W from 0.58 to 0.48 (≈18%). The DP ligament thickness (t) is similar in all the WHAs and pre- and post-annealing conditions. However, these size ratios are unaffected by annealing.

**Figure 2:**
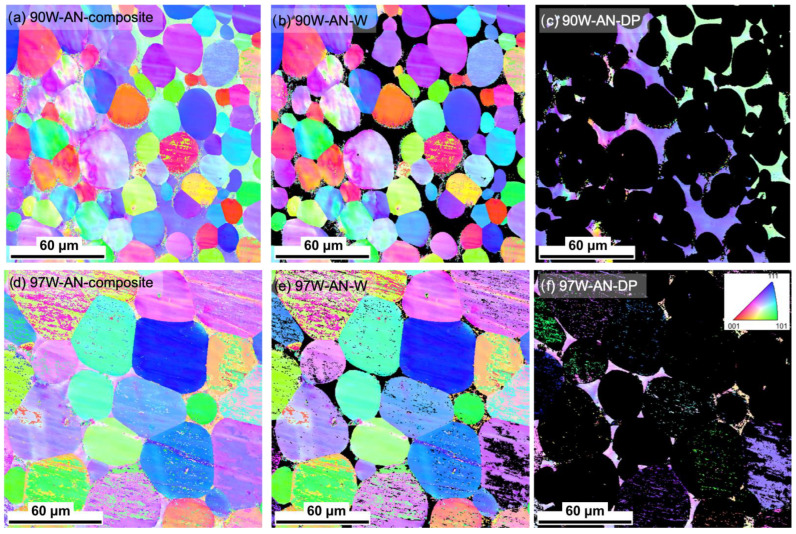
EBSD IPF map showing the random texture orientation for W and DP for 90W (**a**–**c**) and 97W (**d**–**f**) in the AN condition. Here, IPF maps are shown in the left column for the composites, the middle column for W, and the right column for DP for 90W (top row) and 97W (bottom row). The scale bars are all 60 µm. EBSD for the AS condition can be found in [33].

The selective EBSD on the 90 and 97W alloys was characterized to observe possible texture development after annealing. The inverse pole figure (IPF) maps shown in Figure 2 for the annealed 90 and 97W reveal that the W particles remain randomly oriented and the DP is coarse-grained. High misorientation angles (>15°) were not observed in the W particles. These results are similar to the observations on AS 90 and 97W WHAs, reported in [33]. In summary, 1300 °C/24 h annealing has little to no effect on the WHA microstructures examined using SEM, EDS, and EBSD.

### 3.2. Microhardness and Tensile Tests

**Figure 3:**
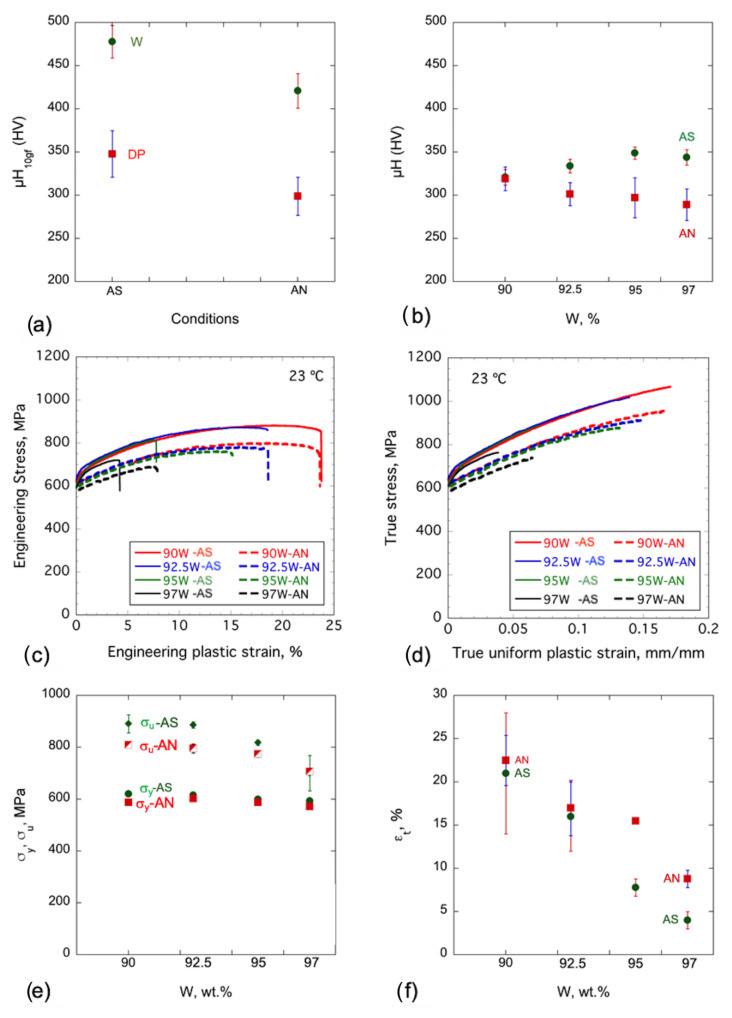
Vickers microhardness HV for AS and AN WHAs for (**a**) individual W particles and DP probed at 10g_f_ load; (**b**) the WHA composites at 500g_f_ load; (**c**) representative RT engineering σ plastic ε_p_ curves; (**d**) true stress-true plastic strain curves; (**e**) 0.2% yield (σ_y_) and ultimate tensile (σ_u_) strengths; and (**f**) total elongations (ε_t_) for AS (green circles) and AN (red squares) WHAs, respectively.

Room temperature Vickers microhardness (HV) measurements were performed on individual W particles, DP regions, and the composite in both the AS and AN conditions. A low 10g_f_ load was used to probe the DP and W particles. Figure 3a shows the AS W and DP HV ≈ 478 ± 19 and 348 ± 27 kg_f_/mm^2^, respectively. After annealing, the HV decreases to 421 ± 20 kg_f_/mm^2^ (≈ 12%) and 299 ± 22 kg_f_/mm^2^ (≈ 14%) in the W particles and the DP, respectively. A 500 g_f_ load was used to measure the WHA composite HV. While the average composite HV increases with W from 321 ± 8 kg_f_/mm^2^ for the 90W to 344 ± 9 kg_f_/mm^2^ for the 97W in the AS conditions, the opposite trend was observed for the AN conditions, decreasing from 319 ± 14 kg_f_/mm^2^ for the 90W to 289 ± 18 kg_f_/mm^2^ for the 97W alloy, as shown in Table 3 and Figure 3b.

Figure 3c shows the representative RT engineering tensile stress–plastic strain (σ−ε_p_) curves, while Figure 3d shows the true stress–true plastic strain curves. Annealing slightly decreases σ_y_ but has a larger effect on σ_u_ due to lower initial strain hardening (Figure 3d). The corresponding σ_y_ and σ_u_ tensile properties are plotted in Figure 3e. The σ_y_ decreases slightly after annealing by <5%. The decrease in σ_u_ is larger up to 92.5W, with a drop of 11%, closes the gap thereafter, and equals 97W due to an increase in σ_t_ (see Table 3 and Figure 3e). Table 3 and Figure 3d also show the average flow stress (σ_fl_) between 0 and 0.1 true plastic strain. Note that the flow stress (σ_fl_) curves are linearly extrapolated for tests that fractured at less than 0.1 true fracture strain. The average σ_fl_ for all the WHAs decreases ≈ 6.4% following annealing. HV depends on, and can be correlated with, σ_fl_, rationalizing the observed HV decreases, while σ_y_ is less affected by annealing.

The total elongation decreases systematically with increasing W in both AS and AN conditions. Annealing has little effect on ε_u_ between 90 and 92.5W but nearly doubles this measure of tensile ductility for the 95 and 97W alloys. Notably, in all cases, the tensile data in the AN condition have lower standard deviations, suggesting that annealing might help to homogenize the microstructure and heal processing damage, which is also an issue for the toughness properties of the as-sintered condition [27].

### 3.3. Room Temperature (RT) Fracture Toughness

**Figure 4:**
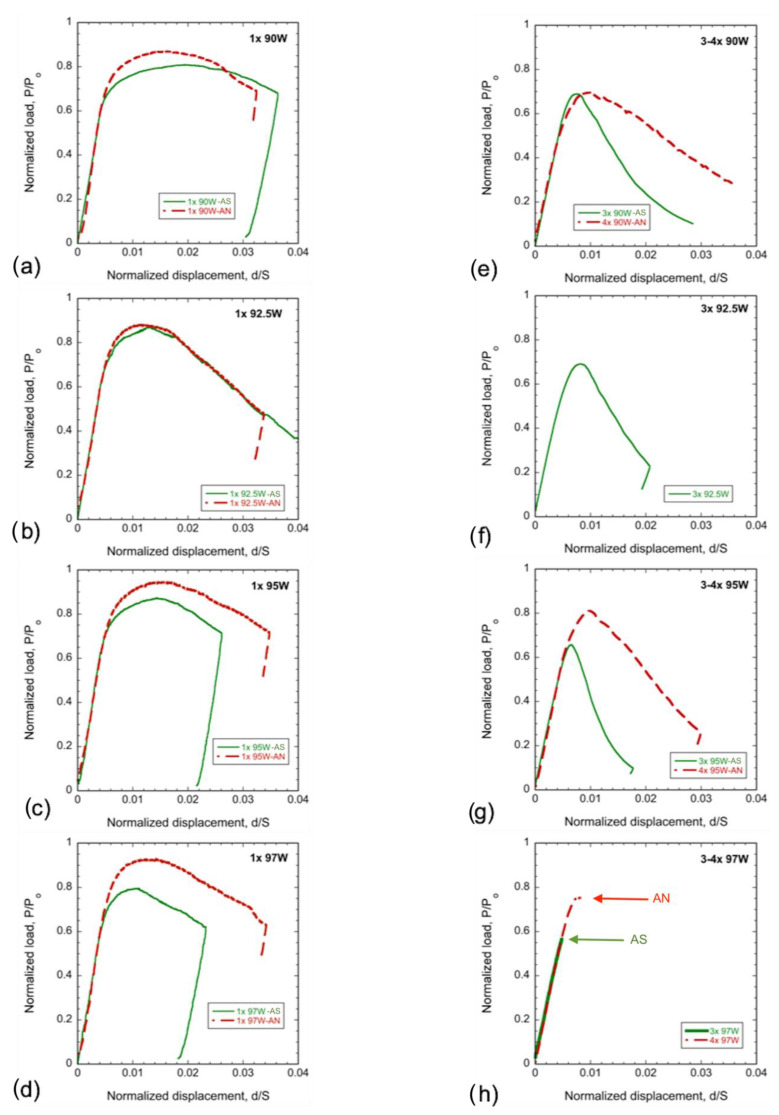
Representative RT normalized load-displacement (P/P_o_-d/S) curves for (**a**–**d**) 1× and (**e**–**h**) 3× AS and 4× AN WHAs. Note, the 4× 92.5W was not annealed. Here, solid green and broken red lines represent the AS and AN conditions, respectively.

Representative normalized load displacement curves, P/P_o_–d/S, for the 3-point bend bar tests are plotted in Figure 4a–d (for 1×) and Figure 4e–h (for 3–4x). Here, P is the load, P_o_ is the limit load, d is the displacement, and S is the bend bar span. As reported previously [14] and shown in Figure 4a–d, all of the AS WHAs 1× specimens show stable crack growth, signaled by gradual post-maximum load drops. The AN WHAs follow a similar P/P_o_–d/S trend with stable crack growth; however, the maximum P/P_o_ is higher than for the AS condition. This is partly due to the decreased limit load (P_o_) for annealed specimens with lower flow stress, which, in this case, is taken as the average of σ_y_ and σ_u_ [27,31,34] (see Table 3). The d at maximum P/P_o_, d_m_, also slightly increased in the AN condition, except at 92.5W.

Figure 4e–h shows the corresponding normalized P/P_o_–d/S curves for the 3× AS and 4× AN WHAs. The 3× AS 90–95W specimens show stable crack growth, while the 3× 97W tests show unstable fracture [27]. The AN 4× specimens also show stable crack growth for the 90 and 95W alloys (note that the 4× 92.5W has not been annealed). In contrast, the 4× 97W AN condition shows only very limited, or no, yielding before fast, unstable fracture (Figure 4h). The 1100 and 1200 °C/1 h annealed 4× 97W specimens also elastically fracture at loads much less than P_o_ (not shown here). As expected, the P/P_o_ for larger specimens is lower than for the 1× specimens due to the larger crack length. Note that in all AN cases, the maximum P/P_o_ is higher than their AS counterparts, and the d/S at maximum P/P_o_ is also larger for cases with stable fracture.

**Figure 5:**
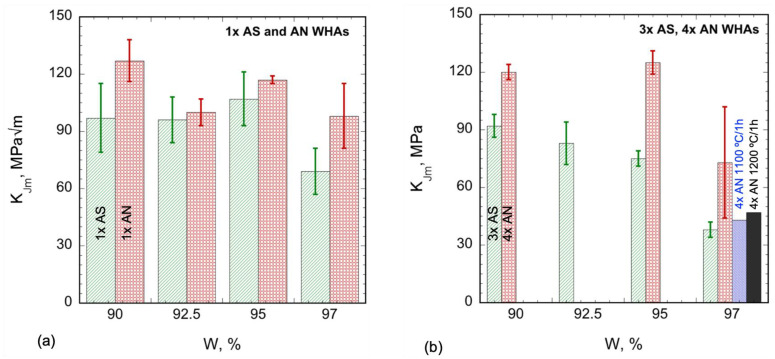
Bar graphs showing the average maximum load toughness K_Jm_ for (**a**) 1× and (**b**) 3−4× WHA as-sintered (AS) and annealed (AN) specimens. Note, all the specimens were annealed at 1300 °C/24 h, except two of the 4× 97W that were annealed at 1100 and 1200 °C/1 h.

The K_Im_ and K_Jm_ values are summarized in Table 4. Note, annealing has little effect on K_Im_ in the 1× specimens (see Supplementary Materials, Figure S3a). However, as shown in Table 4 and plotted in Figure S3a, annealing increases K_Im_ in the 3 and 4× tests at 95 and 97W. Figure 5a plots the 1× K_Jm_ data, showing that annealing increases the average K_Jm_ ≈ 18 MPa√m for all of the WHAs. The corresponding fractional increases are 30, 4, 9, and 42% for the 90, 92.5, 95, and 97W alloys, respectively. The significant effects of specimen size have been previously observed between the 1× and 3× specimens in the AS condition [27]. The size effects appear to be much smaller for the annealed WHAs. As shown in Table 4 and Figure 5b, annealing has a large effect on K_Jm_, with an average increase of 38 MPa√m for the 90, 95, and 97W, representing increases in K_Jm_ of ≈32, 66, and 92%, respectively.

## 4. Discussion

### 4.1. Damage Mechanism for RT Tensile Tests

**Figure 6:**
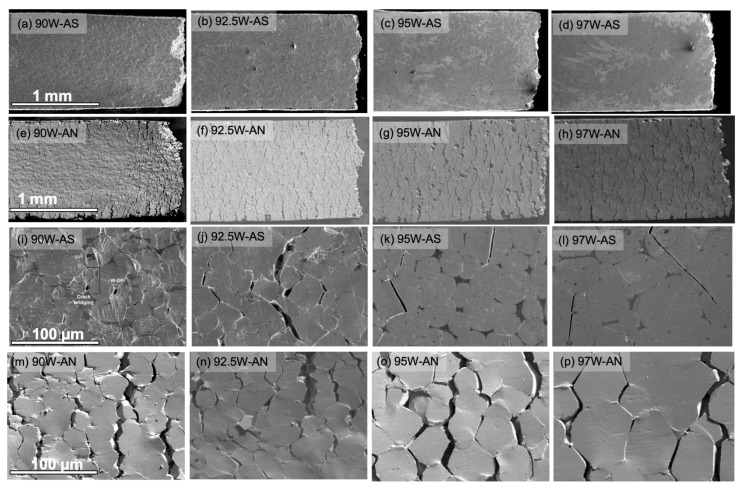
SEM micrographs on the gauge section surface of the tensile specimens for (**a**–**d**) AS and (**e**–**h**) AN WHAs for 90, 92.5, 95, and 97W, respectively. Higher magnification SEM images for respective WHAs are shown in (**i**–**l**) AS and (**m**–**p**) AN conditions. The scale bars for the respective rows are shown in the first column.

Figure 6a–h shows the low magnification SEM micrographs of the tensile gauge section side surface for WHAs in AS (Figure 6a–d) and AN (Figure 6e–h) conditions. Higher magnification SEM images for respective alloys are shown in Figure 6i–l for the AS and Figure 6m–p for the AN conditions. The microcrack damage development is very different in these two conditions. In the AS condition, the microcracks are confined to the region near the fracture surfaces and are largely absent in other parts of the gauge section. In contrast, in the AN condition, a series of arrested microcracks develop over a mm scale distances away from the final fracture surface and in combination span the entire width of the gauge section face.

Corresponding high magnification SEM images of the AS condition show that the W particles are deformed and cleaved, forming microcracks that are arrested and blunted by the DP, especially for the 90 and 92.5W alloys (see Figure 6i,j). The microcracks in the 95 and 97W alloys are sharper and interconnect to span several W particles before failure without affecting the neighboring particles (see Figure 6k,l) responsible for their lower ductility. In the AS conditions, the DP effectively carries and transfers the local microcracked W particle load to nearby intact W regions, which deform before failure. Further, details of tensile test damage development for AS WHAs can be found in Ref. [14].

In contrast, as noted above, the AN WHA condition shows well-distributed microcrack development throughout the gauge length (Figure 6e–h). The higher magnification micrographs for the AN condition reveal the distributed microcracking damage mechanism shifts from localized cleaved W-particle microcrack–microcrack arrest-blunting in the AS condition to more widely distributed W-W, W-DP decohesion, and a DR dominated mechanism in the AN condition (see Figure 6m–p).

**Figure 7:**
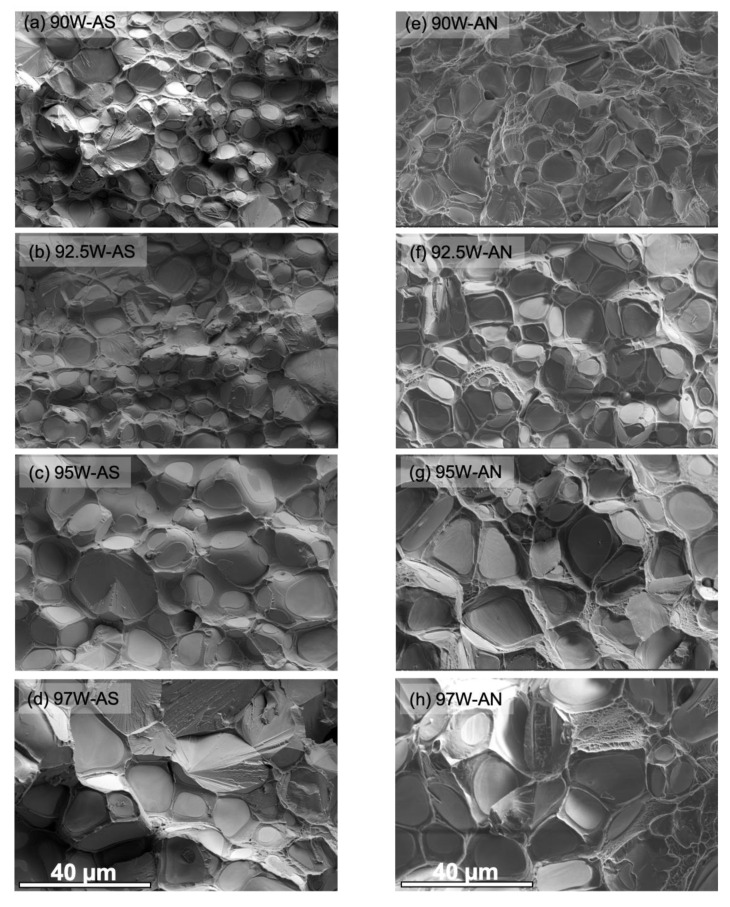
SEM micrographs probed on the fractured face for tensile specimens for (**a**–**d**) AS and (**e**–**h**) AN 90, 92.5, 95, and 97W WHAs, respectively.

The SEM micrographs of the fracture surfaces of the AS and AN WHAs in Figure 7 show all four well-known local failure modes, namely, W–W interparticle fracture (WW), W cleavage (WC), W-NiWFe interfacial debonding (WD), and NiWFe ductile phase rupture (DR) [14]. At 97W, the AS condition shows a higher fraction of WC, whereas more WW and DR are observed in the AN condition.

### 4.2. Fracture Mechanisms in Bend Tests

**Figure 8:**
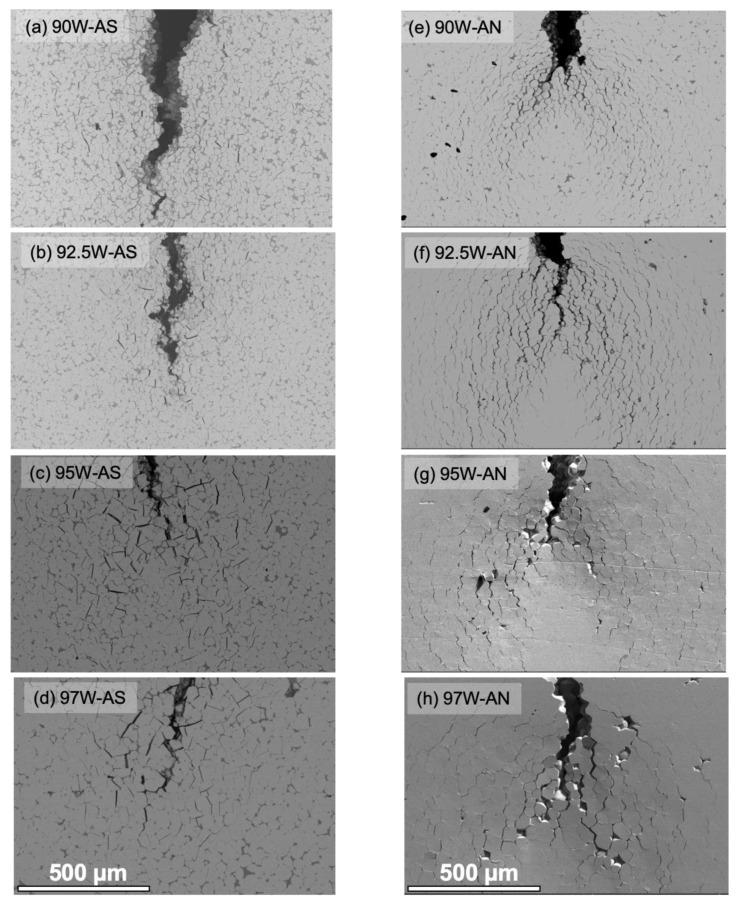
Low magnification SEM images of 1× 90 to 97W propagating cracks in the AS (**a**–**d**, left column) and AN (**e**–**h**, right column) conditions. The scale is 500 µm in all cases.

**Figure 9:**
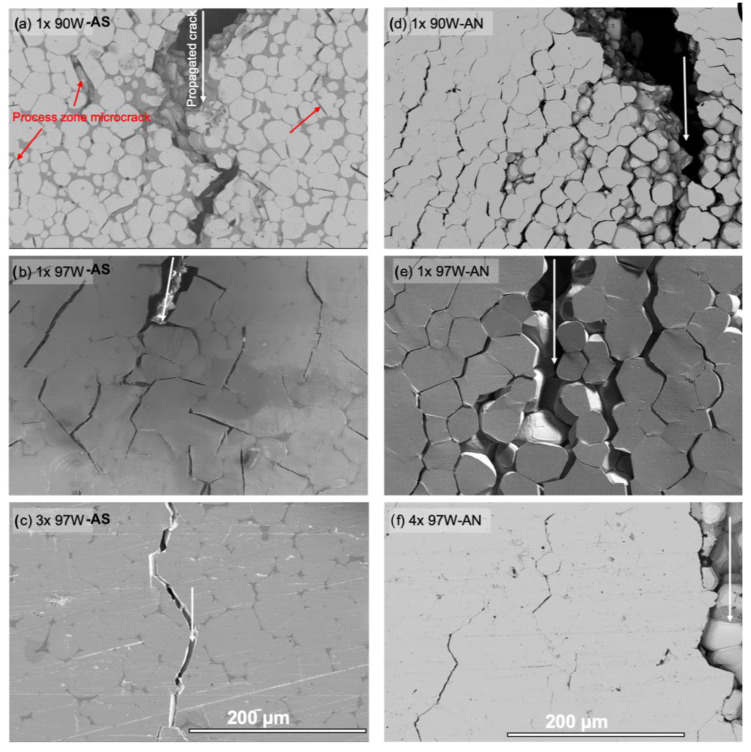
Higher magnification SEM images of 1× 90 to 97W propagating cracks in the AS (**a**–**c**, left column) and AN (**d**–**f**, right column) conditions. Note, (f) is for a fully fractured specimen. The white arrows show the crack propagation direction, and the red arrows show some WC microcracks. The scale is 200 µm in all cases.

Annealing also leads to a major change in the microcracking pattern associated with macrocrack propagation, as illustrated in Figure 8. In contrast to more localized near-tip microcracking in the AS condition, annealing leads to much more and widely distributed microcracking well ahead of the macrocrack and results in crack branching. The shape of the AN condition microcracking pattern reflects the larger plastic zone formed by the loaded macrocrack, with two distinct lobes marking the highest principal normal stresses (see Figure 8e–h). The extensive microcracking following annealing results in enhanced crack tip shielding, leading to a higher toughness. Similar microcracking patterns are observed in the larger 3× and 4× specimens shown in Figure 9.

**Figure 10:**
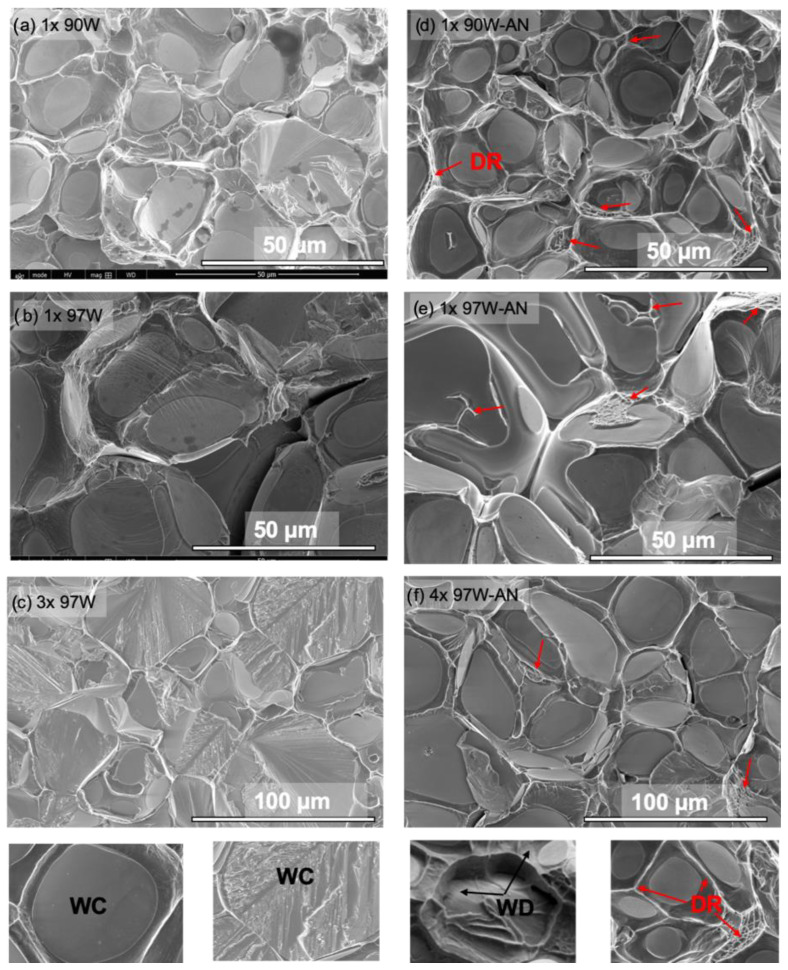
SEM micrographs of fracture surfaces: (**a**) AS 1× 90W; (**b**) AS 1× 97W; (**c**) AS 3× 97W; (**d**) AN 1× 90W; (**e**) AN 1× 97W; (**f**) AN 4× 97W. The bottom row illustrates the nature of local fracture modes.

The local fracture modes are generally similar before and after annealing and for both the 1× and 3 or 4× specimens. One exception is the AS 97W alloy, which experiences a large increase in the W-particle cleavage (WC) from values of 17.6% for 1× to 57.1% for 3× 97W WHAs, as shown in Figure 9 and Figure 10, and Supplementary Materials, Figure S4 and Table S2. In this case, the increase in cleavage is likely due to a combination of higher constraint in the 3× versus the 1× bend bars and the low fraction of DP, allowing earlier linking of planar cleavage microcracks. Further details on the damage mechanism can be found in previous publications [14,27].

## 5. Conclusions

High-temperature 1300 °C/24 h annealing of 90 to 97W WHAs has a minimum effect on the as-sintered (AS) microstructure. The HV decreases in the annealed (AN) condition at higher W contents (HV ≈ 321 ± 8 for AS and ≈319 ± 14 kg_f_/mm^2^ for AN 90W, while for 97W, the HV are 344 ± 9 for AS and 289 ± 18 kg_f_/mm^2^ for AN 97W). However, while annealing has little effect on the corresponding yield strength (≈2 to 5% lower), the uniform and total elongation doubles in 95W (ε_t_ ≈ 8 ± 1% vs. 16 ± 0.3%) and 97W (ε_t_ ≈ 4 ± 1% vs. 8.8 ± 1.0%). With the exception of the 92.5W WHA, annealing increases the 97W elastic–plastic fracture toughness for the smaller WHA up to 42% (69 ± 12 MPa√m vs. 98 ± 17 MPa√m). Annealing also improves the K_Im_ and K_Jm_ for the larger specimens, again especially at higher W contents (from 75 ± 4 MPa√m to 125 ± 6 MPa√m for 95W and 38 ± 4 MPa√m to 73 ± 29 MPa√m for 97W). The size effect between the 1× and 4× specimens is minimal in the AN condition. Given the lower allowable Ni contents with higher RT fracture toughness and tensile ductility, the 1300 °C/24 h annealed 95 and 97W WHAs would be more suitable for the nuclear fusion divertor application.

The microcracking pattern is very different in the AS versus the AN condition. The AS microcracking is more localized to the near-tip process zone of the macro-fatigue crack. In contrast, after annealing, the microcracking is much more widely distributed in the large plastic zone principal stress lobes formed by the loaded fatigue crack. The enhanced microcracking leads to additional dilatational toughening. The local fracture surface damage modes are similar in both the AS and AN conditions, except at 97W. However, the microcracking mechanisms in both the tensile and fracture specimens shift from WC to WW and WD mechanisms in the AN condition. Given the similarity in the AS and AN conditions’ microstructures and strength properties, it seems likely that the effects of annealing are due to increased ductility and a reduction in residual stresses. Thus, XRD studies will be pursued in the near future.

## Figures and Tables

**Table 1:** The nominal compositions and density of the WHAs.

WHAs	W (wt.%)	Ni (wt.%)	Fe (wt.%)	Density (g/cc)
90W	90.27	6.78	2.95	17.05
92.5W	92.48	5.33	2.19	17.58
95W	95.03	3.48	1.49	18.07
97W	97.13	2.01	0.86	18.51

**Table 2:** The W-particle diameter, D_W_, DP surface area %, W–W contiguity, DP thickness, and t/D_W_ for the as-sintered (AS) and 1300 °C/24 h annealed (AN) WHAs.

WHAs	Condition	W particles, D_w_, µm	DP area, %	W–W contiguity, C_w_	DP thickness, t, µm	t/D_W_ µm/µm
90W	AS	17 ± 7	16.1 ± 3.8	0.285	5.9 ± 5.4	0.35
	AN	17 ± 6	18.6 ± 0.8	0.302	5.7 ± 4.7	0.33
92.5W	AS	18 ± 7	11.8 ± 2.2	0.402	4.0 ± 3.7	0.22
	AN	19 ± 7	11.0 ± 0.8	0.408	5.7 ± 6.4	0.3
95W	AS	26 ± 11	10.7 ± 1.3	0.426	5.1 ± 4.1	0.20
	AN	28 ± 11	9.3 ± 0.8	0.405	4.2 ± 3.3	0.15
97W	AS	38 ± 15	6.4 ± 1.5	0.582	4.5 ± 5.0	0.12
	AN	38 ± 14	5.7 ± 0.1	0.477	5.3 ± 4.0	0.14

**Table 3:** RT Vickers microhardness (HV) and tensile properties of 90–97W WHAs.

WHAs	Condition	HV_0.5_ Kg_f_/mm^2^	σ_y_, MPa	σ_u_, MPa	σ_fl_, MPa	ε_u_, %	ε_t_, %
90W	AS	321 ± 8	621 ± 29	891 ± 35	786	18 ± 4	21 ± 7
	AN	319 ± 14	588 ± 12	809 ± 11	730	17.3 ± 1.0	22.5 ± 2.9
92.5W	AS	334 ± 8	616 ± 44	886 ± 12	786	13.5 ± 2.2	16 ± 4
	AN	302 ± 13	603 ± 4	796 ± 12	735	14.1 ± 2.2	17.0 ± 3.2
95W	AS	349 ± 7	600 ± 15	818 ± 10	779	7.3 ± 1	8 ± 1
	AN	279 ± 23	588 ± 9	774 ± 10	718	13.1 ± 1.9	15.5 ± 0.3
97W	AS	344 ± 9	594 ± 27	701 ± 67	708	3.4 ± 1	4 ± 1
	AN	289 ± 18	572 ± 14	707 ± 10	696	7.5 ± 0.9	8.8 ± 1.0

AS = as-sintered, AN = annealed, σ_y_ = 0.2% yield stress, σ_u_ = ultimate tensile strength, σ_fl_ = flow stress, ε_u_ = uniform elongation, ε_t_ = total elongation.

**Table 4:** RT maximum load fracture toughness (K_Im_ and K_Jm,_ in MPa√m) of AS [14,27] and AN WHAs.

Conditions	90W		92.5W		95W		97W	
	K_Im_ (MPa√m)	K_Jm_ (MPa√m)	K_Im_ (MPa√m)	K_Jm_ (MPa√m)	K_Im_ (MPa√m)	K_Jm_ (MPa√m)	K_Im_ (MPa√m)	K_Jm_ (MPa√m)
1× AS	36 ± 4	97 ± 18	39 ± 4	96 ± 12	42 ± 6	107± 14	36 ± 5	69 ± 12
1× AN-1324 ^a^	37 ± 1	127 ± 11	37 ± 1	100 ± 7	38 ± 1	117 ± 2	35 ± 1	98 ± 17
3× AS	52 ± 2	92 ± 6	50 ± 3	83 ± 11	49 ± 1	75 ± 4	38 ± 4	38 ± 4 *
4× AN-1324 ^a^	53 ± 4	120 ± 4	-	-	65 ± 2	125 ± 6	55 ± 3	73 ± 29
4× AN-111 ^b^	-	-	-	-	-	-	43	43 *
4× AN-121 ^c^							47	47 *

* Unstable fracture; 1324 ^a^: 1300 °C/24 h; 111 ^b^: 1100 °C/1 h; and 121 ^c^: 1200 °C/1 h anneal.

## Data Availability

Data will be available upon request.

## Supplementary Materials

The following supporting information can be downloaded at: https://www.mdpi.com/article/10.3390/ma16030916/s1, Figure S1: Low magnification SEM images of 90 to 97W WHAs before ((**a**–**d**), left column: AS) and after ((**e**–**h)**, right column: AN) 1300 °C/24 h annealing. The scale is 200 µm for all cases; Figure S2: EDS-point scan showing DP constituents (i.e., Ni, W, and Fe wt.%) as a function of WHA alloy compositions and annealing conditions; Figure S3: K_Im_ for (**a**) 1× and (**b**) 3-4× WHAs for as-sintered (AS) and annealed (AN) conditions; Figure S4: Local fracture modes for the as-sintered (AS) 3× and annealed (AN) 4× WHAs; Table S1: EDS-point scan results for DP composition variations as a function of WHA alloy contents and annealing conditions; Table S2: The percentage of local fracture features from the WHA toughness fractographs.

## Nomenclature

A_p_	Plastic area under the load-displacement curve, N-m
a	Crack length, mm
B	Specimen thickness, mm
b_o_	Initial unbroken ligaments, mm
d	Load point displacement, mm
D_w_	Diameter of tungsten particle, µm
E	Elastic modulus, GPa
J_e_	Elastic component of J, kJ/m^2^
J_p_	Plastic component of J, kJ/m^2^
J_m_	Measured total J = J_e_ + J_p_ at maximum load, kJ/m^2^
K_Im_	Measured elastic fracture toughness at maximum load = √[J_e_E/(1−ν^2^)], MPa√m
K_Jm_	Measured elastic–plastic fracture toughness at maximum load, MPa√m
P	Load, N
P_m_	Maximum load, N
P_o_	General yield limit load, N
S	Span, mm
σ_fl,_ σ_o_	Flow stress, MPa
σ_u_	Ultimate strength, MPa
σ_y_	Yield stress, MPa
ε	Tensile elongation, %
ν	Poisson’s ratio
3PB	Three-point bend specimen
AN	Annealed
AS	As-sintered
ASDEX	Axially symmetric divertor experiment
ASTM	American society for testing and materials
DP	Ductile phase
DR	Ductile rupture
EBSD	Electron backscatter diffraction
EDM	Electrical discharge machining
EDS	Energy dispersive spectroscopy
IPF	Inverse pole figure
LPS	Liquid-phase sintering
MW/m^2^	Megawatt per meter square
RT	Room temperature
SEM	Scanning electron microscopy
W	Tungsten
WC	Tungsten particle cleavage
WD	Tungsten-DP decohesion
WW	Tungsten particle–particle fracture
WHA	Tungsten heavy alloy
