# Supplementary material for: Improving the Fracture Toughness and Ductility of Liquid-Phase Sintered WNiFe Tungsten Heavy Alloys by High-Temperature Annealing
Source: Materials (Basel). 2023 Jan 18;16(3):916. doi: 10.3390/ma16030916 (PMC9917838; doi:10.3390/ma16030916)
Supplement: Supplementary file 1 [file materials-16-00916-s001.zip › materials-2080563-supplementary.pdf]

materials

MDPI

Supplemental Figures, SF

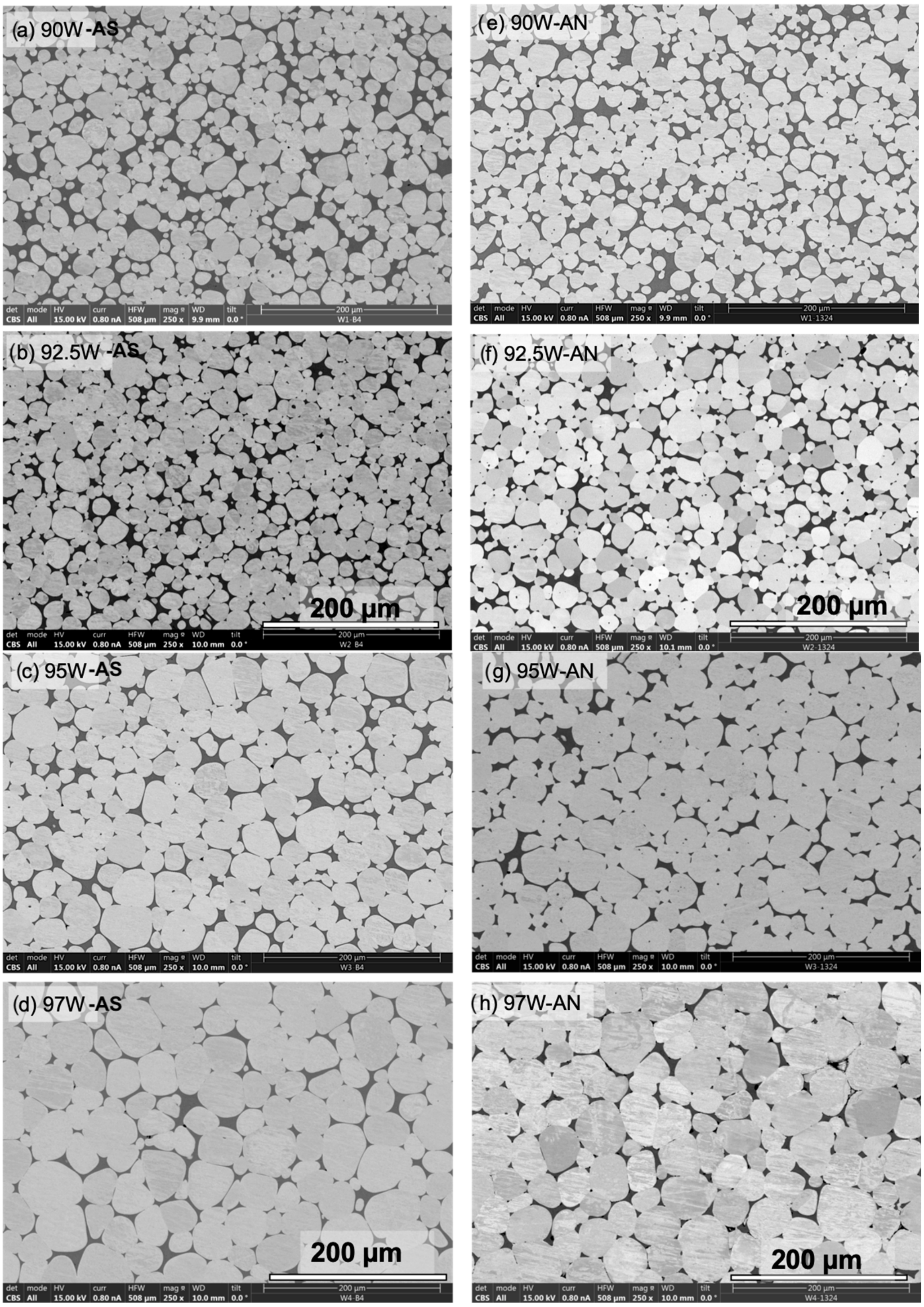

Figure S1. Low magnification SEM images of 90 to 97W WHAs before (a-d, left column: AS) and after (e-h, right column: AN) 1300 °C/24 h annealing. The scale is 200 µm for all cases.

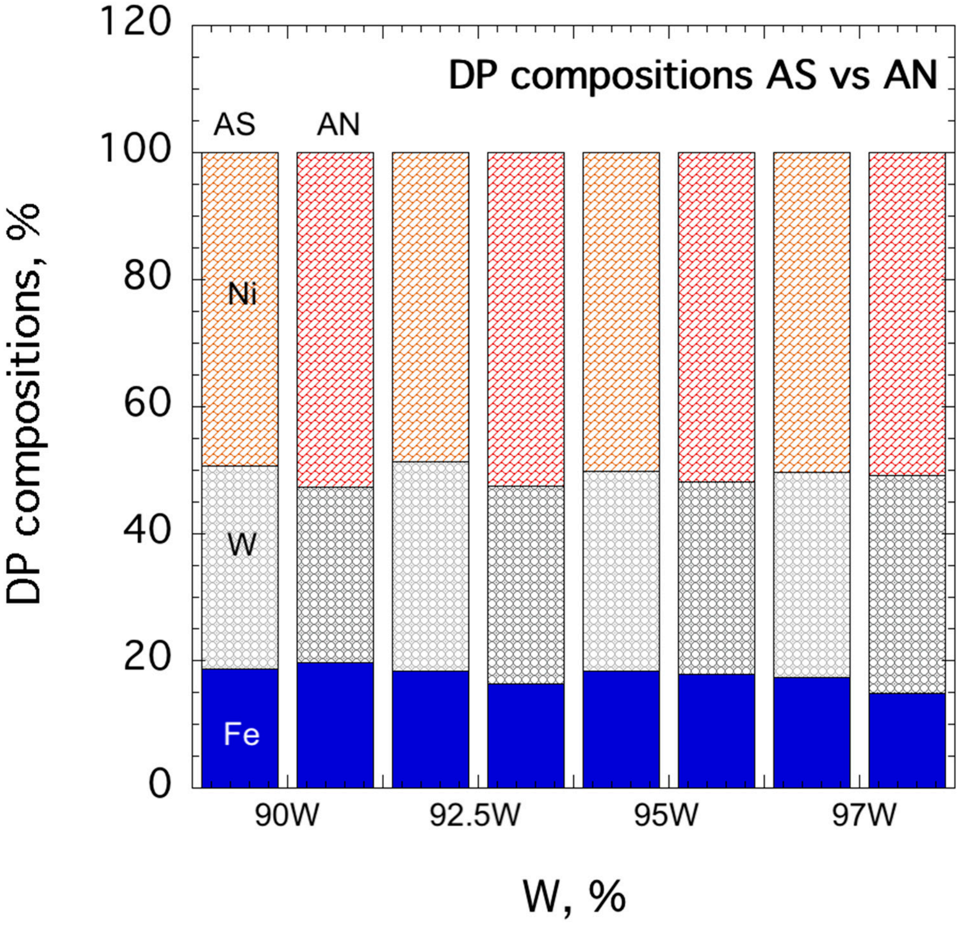

Figure S2. EDS point scan showing DP constituents (i.e., Ni, W, and Fe wt.%) as a function of WHA alloy compositions and annealing conditions.

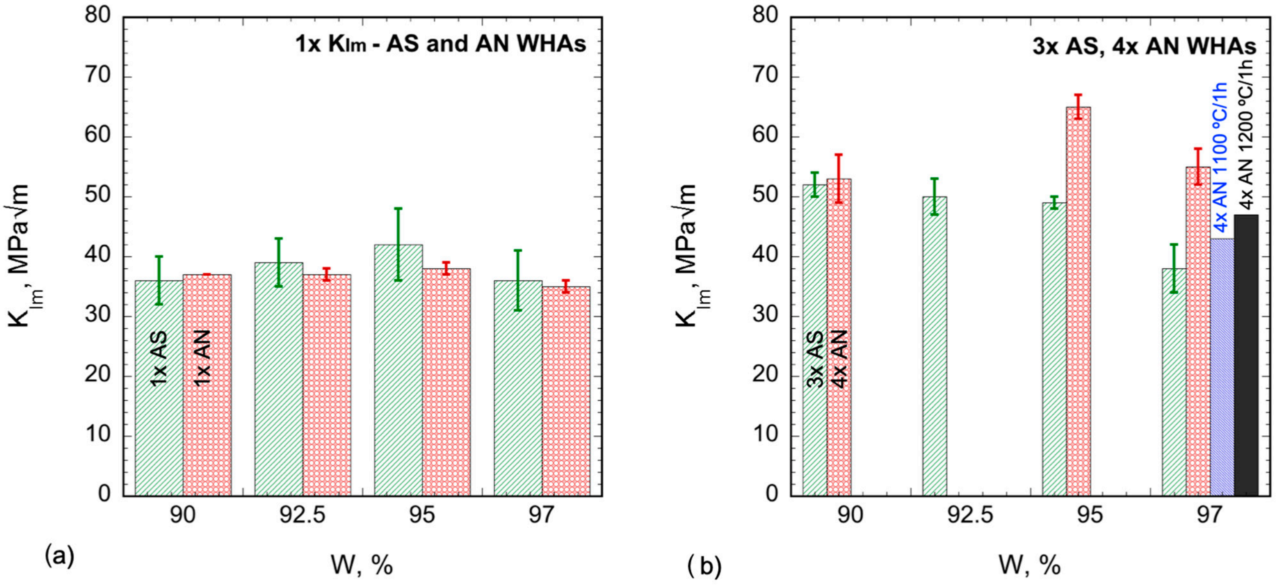

Figure S3. K_{Im} for a) 1x; an, b) 3-4x WHAs for as-sintered (AS) and annealed (AN) conditions.

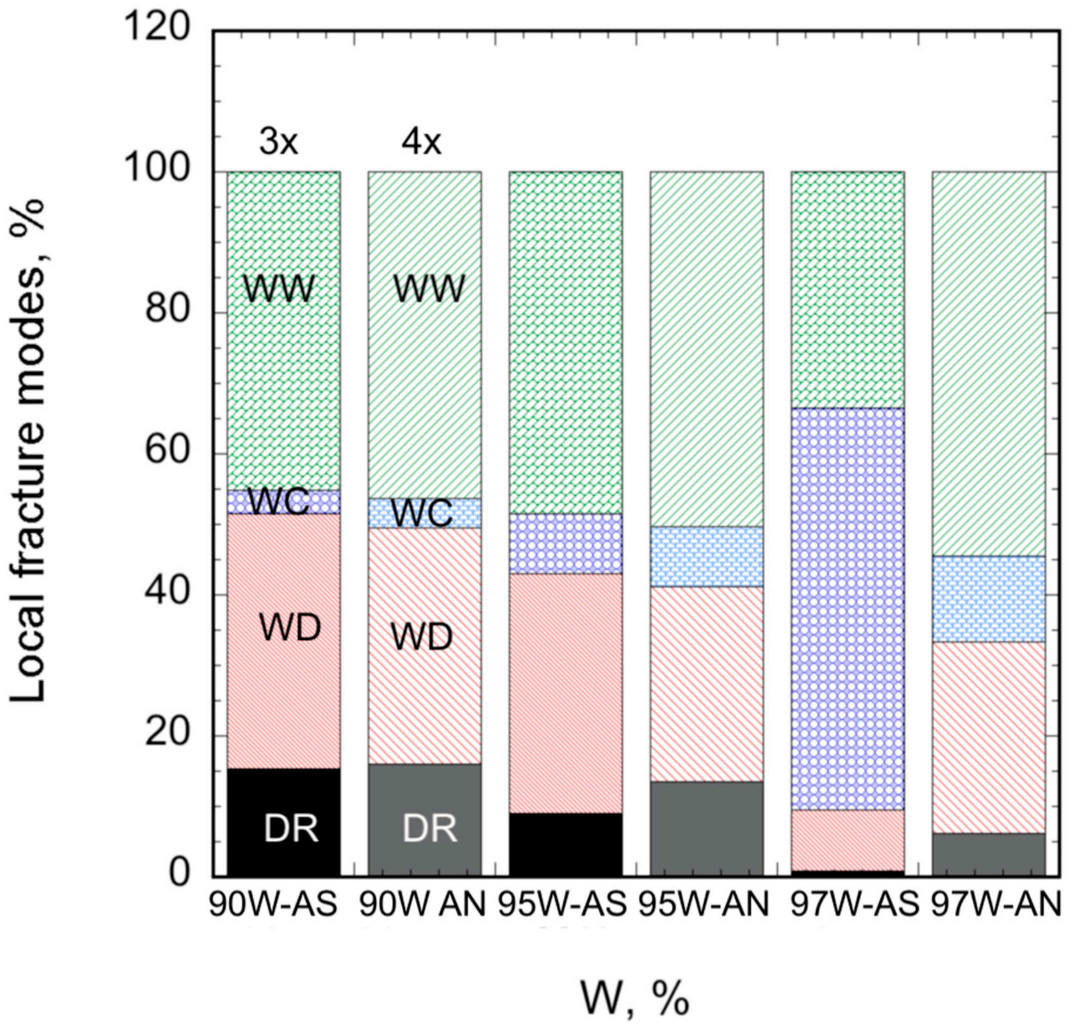

Figure S4. Local fracture modes for the as-sintered (AS) 3x and annealed (AN) 4x WHAs.

Supplemental Tables, ST

Supplemental Table S1: EDS point scan results for DP composition variations as a function of WHA alloy contents and annealing conditions.

WHAs	Condition	Ni, wt. %	W, wt. %	Fe, wt. %
90W	AS	49.3 ± 0.4	32. 0 ± 0.4	18.7 ± 0.2
	AN	52.7 ± 0.9	27.6 ± 1.1	19.7 ± 0.5
92.5W	AS	48.7 ± 0.7	32.9 ± 1.2	18.4 ± 0.6
	AN	52.4 ± 0.2	31.2 ± 0.3	16.4 ± 0.3
95W	AS	50.2 ± 0.7	31.5 ± 0.9	18.3 ± 0.3
	AN	51.9 ± 1.2	30.2 ± 1.4	17.9 ± 0.5
97W	AS	50.3 ± 0.6	32.3 ± 0.7	17.4 ± 0.3
	AN	50.8 ± 0.5	34.3 ± 1.5	14.9 ± 1.5

Supplemental Table S2: The percentage of local fracture features from the as-sintered (AS) and annealed (AN) WHA toughness fractographs.

WHAs	Condition	WW (%)	WC (%)	WD (%)	DR (%)
1x 90W	AS	31.7	12.0	44.1	12.2
	AN	35.4	8.8	45.7	10.1
1x 92.5W	AS	37.1	10.9	40.5	11.5
	AN	47.8	7.7	37.2	7.3
1x 95W	AS	42.6	13.5	32.8	11.1
	AN	45.2	13.7	33.5	7.6
1x 97W	AS	67.2	17.6	10.8	4.4
	AN	56.2	22.5	16.9	4.4
3x 90W	AS	45.1	3.4	36.2	15.3
4x 90W	AN	46.3	4.1	33.6	16.0
3x 95W	AS	48.4	8.6	34.0	9.0
4x 95W	AN	50.3	8.6	27.5	13.6
3x 97W	AS	33.4	57.1	8.7	0.8
4x 97W	AN	54.5	12.1	27.3	6.1
